# Indoor multiuser visible light communication systems using Hadamard-coded modulation

**DOI:** 10.1098/rsta.2019.0183

**Published:** 2020-03-02

**Authors:** Jie Lian, Mohammad Noshad, Maïté Brandt-Pearce

**Affiliations:** a Northwestern Polytechnical University, School of Marine Science and Technology, Xi’an 710072, People’s Republic of China; b VLNcomm, Charlottesville, VA 22911, USA; c University of Virginia, Charlottesville, VA 22904, USA

**Keywords:** Hadamard-coded modulation, visible light communications, multiuser systems, optical wireless communications

## Abstract

Visible light communications (VLC) is a short-range optical wireless communication technology that uses light-emitting diodes (LEDs) as lighting devices and data transmitters. This paper describes a multiuser VLC system using Hadamard-coded modulation (HCM) for indoor data transmission. Considering the peak transmitted power limit of the LEDs, a DC-reduced HCM (DCR-HCM) is used to reduce the nonlinear clipping distortion. Since the Hadamard codewords have different bandwidth requirements for a given symbol rate, they can be assigned to users with varying hardware capabilities. Optimally assigning codewords to users is found to significantly improve the average throughput, up to twice higher than a random assignment for a typical scenario. When the number of active users is less than the size of the Hadamard matrix used, more than one codeword can be assigned per user, which further improves the throughput. This paper also examines a scenario where multiple lamps in an indoor space transmit the same data. Since the time of arrival for the received signals emitted from different lamps is different, the Hadamard codes received are no longer orthogonal, resulting in multiple access interference and inter-chip interference. The number of acceptable codewords is computed based on the specific interference experienced in different parts of the indoor space. The spatial distribution of the maximum throughput is also simulated, showing that the ratio of the maximum to the minimum data rate can be as high as 10 when considering the entire area of a typical indoor room.

This article is part of the theme issue ‘Optical wireless communication’.

## 1. Introduction

Visible light communications (VLC), using light-emitting diodes (LEDs) as transmitters, has recently garnered significant research attention from industry and academia due to its advantages over radio frequency (RF) communications [1,2]. VLC systems are immune to electromagnetic interference, are safe for human health, have high energy efficiency, and can provide high security and high-speed wireless access [3].

LEDs working as lighting devices have been widely deployed, creating an opportunity for a ubiquitous wireless network using VLC. Due to the properties of the LEDs (non-coherent light), and the photodetectors (PDs) needed at the receivers, intensity modulation and direct detection (IM/DD) must be used, which requires the transmitted signals to be non-negative and real-valued. Therefore, traditional modulation schemes used in RF systems cannot be adopted directly in VLC. Because of the light reflected off obstacles, the indoor channel is dispersive, which limits the system throughput. The overall VLC channel is further bandlimited by the slow rise-time of lighting LEDs [4,5]. High spectral-efficiency intensity-modulation techniques are therefore of great interest to VLC designers.

Orthogonal frequency division multiplexing (OFDM), a multi-carrier modulation scheme that was originally designed for RF systems, has been proposed and applied to optical systems due to its high spectral-efficiency and resistance to inter-symbol interference (ISI) [6,7]. Many advanced optical OFDM schemes have been designed for VLC systems, such as DC-biased optical OFDM (DCO-OFDM), asymmetrically clipped optical OFDM (ACO-OFDM), flip-OFDM, and unipolar-OFDM (U-OFDM) [7–9]. However, optical OFDM was found in [10] to be inferior to pulsed techniques when the bandwidth is severely limited; *M*-ary pulse amplitude modulation (*M*-PAM) using equalization and pulse shaping provides better performance than optical OFDM [10,11].

Hadamard-coded modulation (HCM) is a pulsed modulation format that uses orthogonal Hadamard codes instead of the orthogonal subcarriers in OFDM to modulate the data [12]. It thus benefits from the advantages of both schemes. HCM can attain high spectral efficiencies using *M*-PAM and yet has a lower peak to average power ratio (PAPR) than OFDM techniques. It thus experiences less nonlinear distortion due to the power-constrained LEDs [12]. This paper explores the advantages of using HCM in multiuser VLC systems where users may have varying computational resources.

Providing multiple users with a reliable high data rate is a key performance criterion for wireless communication systems. For VLC systems, code division multiple access (CDMA), time division multiple access (TDMA) and orthogonal frequency division multiple access (OFDMA) can be applied to support numerous users [13–15]; however, in practical scenarios, these multiple access techniques are not flexible enough to transmit data to users with different processing abilities. Using the physical location of the users, space division multiple access (SDMA) can increase diversity in VLC systems [16]. Multiple-input and multiple-output (MIMO), CDMA, TDMA and OFDM techniques can be coupled with SDMA to further improve the system throughput [17].

For Internet of things (IoT) applications, users connected to the network may have different hardware capabilities, depending on the equipped devices. In this paper, we propose to use HCM for multiple users with different processing speeds, i.e. different bandwidth capabilities. Since Hadamard codewords naturally have different bandwidth requirements, they can be optimally attributed to users based on their processing abilities. Low-bandwidth codewords can be assigned to users with low-bandwidth transceivers to minimize ISI.

In this paper, several practical aspects of implementing HCM in a multiuser environment are explored. HCM is a multi-level pulsed modulation scheme, and a larger number of levels requires a more complicated transmitter structure. A DC-reduced HCM (DCR-HCM) is proposed in [12] to reduce both the risk of peak power clipping distortion and high complexity in the transmitter structure. We discuss and compare DCR-HCM and conventional HCM systems. When the number of users is less than the order of the Hadamard code, more than one codeword can be assigned to each user, and the throughput can be enhanced. A multi-lamp scenario where the transmitters send the same data is then explored; the effects of the signal time of arrival difference from spatially separated lamps are analysed and discussed. The received Hadamard codewords are no longer orthogonal, and multiple access interference and inter-chip interference can result, affecting the usability of individual codewords. This paper presents the Hadamard codewords usability distribution and the maximum throughput achievable in a typical indoor environment.

The remainder of the paper is organized as follows. Section 2 describes the multiuser HCM system, which includes models of the channel and the transmitted and received signals. Section 3 presents an HCM MAC layer design for multiple access suitable for single-lamp and multi-lamp scenarios, including analytical and simulation results. The paper is concluded in §4.

## 2. Multiuser Hadamard-coded modulation system

In this section, the model of the proposed indoor multiuser HCM system is described, which includes the wireless channel, transmitted and received signals and the DC-reduced HCM.

### (a) Indoor channel model

In indoor VLC systems, the channel impulse response consists of line-of-sight (LOS) and non-LOS (NLOS) parts. The LOS signal is transmitted from the LED and received directly at the receiver. The NLOS component is caused by reflections of the light off the walls, furniture, ceiling, and other reflective surfaces. Due to these reflections, the indoor VLC channel is dispersive and frequency selective, which degrades the performance of VLC systems. The impulse response can be obtained by using a ray-tracing algorithm [18]. The received signal power depends on the beamwidth of the light source, propagation distance, incident angle, and irradiation angle following the Lambertian rule. The channel loss can be represented as [18],

2.1 $$h=\frac{A_{r}\cos\phi }{2\pi L^{2}}(m+1)\cos^{m}\psi ,$$

where *ϕ* and *ψ* are the incident and radiation angles, respectively. The beamwidth of the LED determines the transmitter’s Lambertian mode, *m*. *L* represent the propagation distance, and *A*_*r*_ is the effective area of the receiver’s PD. LEDs are low-pass devices due to their slow rise-time (the bandwidth of commercial white lighting LEDs is limited to around a few tens of MHz) [5]. The overall channel response can be determined by combining the transmitter and channel responses with the receiver’s low-pass filter, which is a function of its sampling rate. In general, the impulse response of the overall VLC channel can be modelled as a first-order low-pass filter [19].

### (b) Hadamard-coded modulation transmitted signal model

The transmitted HCM signal model is described in this section. Hadamard codes are binary {0, 1} sequences that can be used to modulate data by using a fast Walsh–Hadamard transform (FWHT). To simplify the notation, we analyse the signal in one HCM symbol.

**Figure 1.**
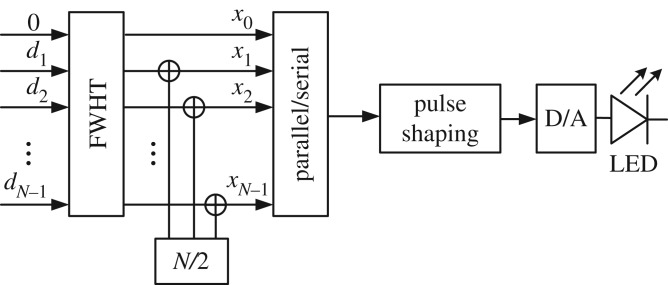
Block diagram of a transmitter in an HCM system.

HCM can be used to support multiple users in a way that is similar to CDMA: the data intended for each user is modulated by one or more Hadamard codewords. As shown in figure 1, the transmitted signals for the multiple users are input to the FWHT. The *M*-ary pulse amplitude modulated (*M*-PAM) symbols from data stream *k* are labelled *d*_*k*_, where *d*_*k*_ ∈ {0, 1/(*M*_*k*_ − 1), 2/(*M*_*k*_ − 1), …, 1}, *k* = 1, 2, …, *K*, and *M*_*k*_ is the modulation constellation size for data stream *k*. *K* represents the number of data streams or active users. After modulation, the HCM signal, **x** = (*x*_0_, *x*_1_, …, *x*_*N*−1_), can be written as

2.2 $$\text{x}=\frac{\beta }{N}\left(\text{d}({\text{H}}_{N}-{\bar{\text{H}}}_{N})+\frac{N}{2}[0,1,1,\ldots ,1]\right),$$

where *β* represents the modulation index that controls the signal scale. **H**_*N*_ is the binary Hadamard matrix of order *N* [20], and ${\bar{\text{H}}}_{N}$ is its binary complement. Since the elements in the first codeword of the Hadamard matrix are all ones, it is not used for modulation; therefore, *N* − 1 data streams can be supported, so that **d** = (0, *d*_1_, *d*_2_, …, *d*_*N*−1_). To avoid transmitting negative signals, a constant bias of *N*/2 is added.

The parallel vector **x** that contains the combined modulated data is serialized and transmitted. Based on the central limit theorem (CLT), each sample of the resulting time series for large *N* can be modelled as an independent Gaussian distributed random variable with mean *μ*_*x*_ = (*N*/2) and variance $\sigma_{x}^{2}=(\beta^{2}/N^{2})\sum_{i=1}^{N}{\hat{\sigma }}_{i}^{2}$, where ${\hat{\sigma }}_{i}^{2}$ represents the variance of *d*_*i*_ [12].

In this paper, we wish to explore the advantages that HCM might have over other modulation schemes for applications such as IoT, where some users may have a low-bandwidth transmitter or receiver because of limited clock speeds. A lower-order FWHT can be used to modulate or demodulate a specific subset of the codewords identified as having a smaller bandwidth. Alternately, a simple code modulation at the transmitter and code matched-filter at the receiver suffices for a single codeword. Thus, using HCM in a multiuser environment, users with lower bandwidth can use a low-complexity and low-cost transmitter or receiver structure to detect narrow bandwidth HCM codes instead of using the full-scale FWHT.

**Figure 2.**
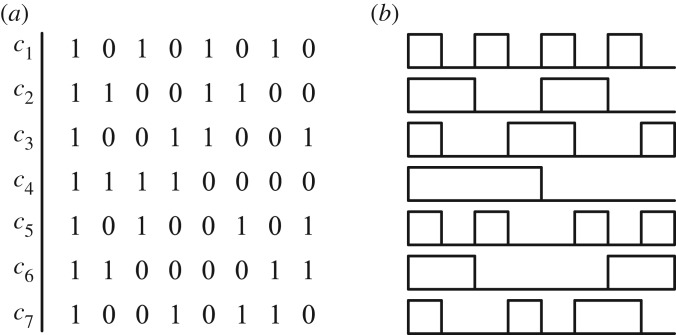
Hadamard codewords with an order of 8. (*a*) Binary codewords, (*b*) waveforms of the Hadamard codewords before transmitter filtering.

Figure 2*a* shows a Hadamard code matrix of size *N* = 8 stripped of its first row; the codewords *c*_1_ to *c*_7_ are used to modulate seven data streams. Figure 2*b* shows the 8-chip waveforms corresponding to the codewords. As is evident from the figure, different codewords require different bandwidths. For example, codeword *c*_4_ requires half of the bandwidth of *c*_6_. The normalized required bandwidth for the Hadamard codewords of order *N* is given in table 1; to make the results independent of the chip rate, we normalize the highest bandwidth to one. From the table, half of the codewords require the highest bandwidth, while the other half can use lower bandwidth transceivers. From this table, only one codeword has the lowest bandwidth, which is a factor 2/*N* smaller than the maximum bandwidth required.

**Table 1.** Normalized bandwidth required for HCM codewords.

HCM codeword	normalized required bandwidth	number of codewords
*c*_1_, *c*_3_, *c*_5_, …, *c*_*N*−1_	1	*N*/2
*c*_2_, *c*_6_, *c*_10_, …, *c*_*N*−2_	1/2	*N*/4
⋮	⋮	⋮
$c_{\frac{N}{4}}$, $c_{\frac{3N}{4}}$	4/*N*	2
$c_{\frac{N}{2}}$	2/*N*	1

Lighting LEDs emit an optical power that is nonlinearly related to their drive current, especially near their peak power limit. The signal beyond the peak power of the LED can be modelled as being hard clipped. We model the LED’s nonlinearity as a piece-wise function, given as

2.3 $$\varphi (x)=\left\{ \begin{array}{ll} P_{\text{max}}, & x\geq P_{\text{max}}\\ x, & 0<x<P_{\text{max}}\\ 0, & x\leq 0 \end{array}\right.,$$

where *P*_max_ represents the value of the peak power of the LED. The modulation index, *β*, shown in (2.2), can be optimally chosen to trade off the clipping distortion and signal power. Alternatively, an LED array containing multiple small on–off-modulated LEDs can be used to represent an analogue waveform, adding circuit complexity but avoiding the LED nonlinear effects.

### (c) Hadamard-coded modulation receiver processing

In this section, the received signal is first represented by using the full-sampling-rate model for all users. We then address the effect of subsampling available to users assigned a low-bandwidth codeword.

A PD at the receiver is first used to convert the received optical power to an electrical signal with a conversion rate, *ρ*, called the responsivity. Without loss of generality, the responsivity is set to unity in all subsequent equations. The sampled electrical signal vector in one HCM symbol, **y** = (*y*_0_, *y*_1_, …, *y*_*N*−1_), can be written as

2.4 $$\text{y}=\rho \cdot \text{h}*\varphi (\text{x})+\text{n}+{\text{n}}_{\text{clip}},$$

where **h** = (*h*[0], *h*[1], …, *h*[*N* − 1]) is the discrete-time version of the system impulse response that includes the effects of the transmitter, channel, and receiver. * denotes the discrete-time convolution operation. The additive noise at the receiver in one HCM symbol, **n** = (*n*[0], *n*[1], …, *n*[*N* − 1]), includes the thermal and background shot noise [2] and can be modelled as an independent Gaussian random vector with zero mean. The variance of the noise samples depends on the receiver bandwidth, *B*, and noise power spectral density, *N*_0_, and can be calculated as $\sigma_{n}^{2}=N_{0}B$. **n**_clip_ denotes the clipping-induced noise that is modelled as an additive Gaussian noise with variance [12]

2.5 σclip2=(12)N∑k=⌈N(Pmax/β)⌉N(kβN−Pmax)2(Nk),

where ⌈x⌉ is the smallest integer larger than *x*.

**Figure 3.**
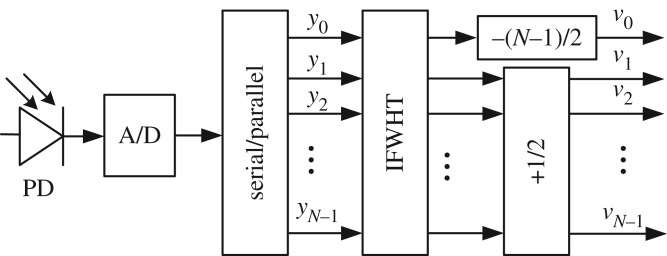
A block diagram of a receiver in an HCM system.

When all the data streams are used for one user, an inverse FWHT (IFWHT) is used to decode the data at the receiver, as shown in the block diagram in figure 3. After this step, the decoded signal vector, **v** = (*v*_0_, *v*_1_, …, *v*_*N*−1_), can be obtained as

2.6 $$\text{v}=\text{y}({\text{H}}_{N}^{\text{T}}-{\bar{\text{H}}}_{N}^{\text{T}})+\frac{1}{2}[1-N,1,1,\ldots ,1].$$

For the multiple user case, if only one data stream is needed at a particular user, its receiver structure can be simplified; only the codeword modulating the intended data is used for demodulation instead of using the IFWHT. Both methods yield the same results.

When a low-bandwidth codeword is assigned to a user with a slower sampling rate, a lower-order Hadamard matrix can be used at the receiver with no performance loss. No interference is introduced as long as the correct Hadamard codewords are used for demodulation. Using the slowest sampling rate needed with the lowest order FHWT can save significant processing power in resource-limited devices. If the size of the Hadamard matrix is increased to support more users, the bandwidth requirements (or, equivalently, the sampling rate requirements) for an already-assigned codeword do not change. For example, when *N* = 4, codeword *c*_2_ is {1 1 0 0}; this codeword at twice the sampling rate becomes {1 1 1 1 0 0 0 0} when *N* = 8, but its bandwidth is unchanged. This gives the HCM-based multiple access approach a huge flexibility advantage as the code size can be adaptively changed without disrupting existing connections.

#### (i) System performance for an ideal channel

It is informative to examine the ideal channel case to understand the system limitations when the transmission bandwidth is larger than needed for the symbol rate used. The decoded vector in this case can be rewritten as

2.7 $$\text{v}=\frac{r\beta }{N}\text{d}+\tilde{\text{n}}+{\tilde{\text{n}}}_{\text{clip}},$$

where *r* represents the clipping coefficient that describes the power loss due to clipping. Using the fact that **x** can be modelled as approximately Gaussian, the clipping coefficient, *r*, can be calculated from the Bussgang theorem [21] using

2.8 $$\begin{array}{rl} r & =\frac{1}{\sigma_{x}^{3}\sqrt{2\pi }}\int_{-\infty }^{\infty }x\varphi (x)\exp\left(-\frac{{\left(x-\mu_{x}\right)}^{2}}{2\sigma_{x}^{2}}\right)\text{d}x\\ & =1-\frac{1}{2}\left(\text{erfc}\left(\frac{-\beta +2P_{\text{max}}}{\sqrt{(8\beta^{2}\sigma_{d}^{2})/N}}\right)+\text{erfc}\left(\frac{\beta +2P_{\text{max}}}{\sqrt{(8\beta^{2}\sigma_{d}^{2})/N}}\right)\right), \end{array}$$

where $\text{erfc}(x)=(2/\sqrt{\pi })\int_{x}^{\infty }\exp(-u^{2})\;\text{d}u$.

The effective noise vectors after the IFWHT operation are denoted as $\tilde{\text{n}}=(\text{n}/N)({\text{H}}_{N}^{\text{T}}-{\bar{\text{H}}}_{N}^{\text{T}})$ and ${\tilde{\text{n}}}_{\text{clip}}=({\text{n}}_{\text{clip}}/N)({\text{H}}_{N}^{\text{T}}-{\bar{\text{H}}}_{N}^{\text{T}})$ in (2.7). $\sigma_{d}^{2}$ represents the variance of *d*_*k*_. Therefore, after HCM demodulation at the receiver, the bit error rate for the *k*th user using *M*-PAM HCM for non-dispersive channels can be approximately calculated from [22] as

2.9 $${\text{BER}}_{k}\approx \frac{M_{k}-1}{2M_{k}\log_{2}M_{k}}\cdot \text{erfc}\left(\sqrt{\frac{3}{\sqrt{2}(M_{k}^{2}-1)}\frac{r^{2}\beta^{2}}{N(\sigma_{n}^{2}+\sigma_{\text{clip}}^{2})}}\right),$$

which is a standard approximation of the BER for *M*-PAM.

#### (ii) System performance for a dispersive channel

A dispersive channel introduces ISI between HCM symbols and interchip interference (IChI) within the codewords; in this paper we ignore the ISI, assuming a large enough *N* is used to make the interference between adjacent symbols relatively small. (If not, a cyclic prefix or guard-time between symbols can be used as in OFDM.) The received signal vector after the IFWHT operation can be represented as in [12],

2.10 $$\text{v}=r\beta ({\text{H}}_{N}-{\bar{\text{H}}}_{N})\text{F}({\text{H}}_{N}-{\bar{\text{H}}}_{N})\frac{2\text{d}-1}{2N}+\frac{\beta }{2}+\tilde{\text{n}}+{\tilde{\text{n}}}_{\text{clip}},$$

where $\text{F}=\sum_{\ell }h[\ell ]{\text{I}}^{\left(\ell \right)}$ and **I**^(ℓ)^ represents the ℓth right cyclic-shifts of the identity matrix. After applying a minimum mean square error (MMSE) equalizer, **W** = (**w**_0_, **w**_1_, …, **w**_*N*−1_)^*T*^, the filtered signal vector is obtained as

2.11 $$\hat{\text{d}}=\text{W}\left(\text{v}-\frac{\beta }{2}\right)+\frac{1}{2},$$

where $\hat{\text{d}}={\left({\hat{d}}_{0},{\hat{d}}_{1},\ldots ,{\hat{d}}_{N-1}\right)}^{T}$ is an MMSE estimate of the data vector **d**. For the multiuser scenario using *M*-PAM, the datum for stream *k* is obtained by appropriately thresholding ${\hat{d}}_{k}$, as in a standard *M*-PAM demodulator.

The optimal MMSE filter **W** can be found by minimizing the trace of $E\{(\text{d}-\hat{\text{d}}){\left(\text{d}-\hat{\text{d}}\right)}^{T}\}$, where *E*{ · } represents the expected value. According to [20], the optimum **W** is given by

2.12 $$\text{W}={\text{C}}_{\text{dv}}{\text{C}}_{\text{v}}^{-1},$$

where **C**_**dv**_ and **C**_**v**_ are

2.13 $$\begin{array}{rl} & {\text{C}}_{\text{dv}}=\frac{\beta }{4N}({\text{H}}_{N}-{\bar{\text{H}}}_{N})\text{F}({\text{H}}_{N}-{\bar{\text{H}}}_{N})\\ \;\text{and}\; & {\text{C}}_{\text{v}}=\frac{\sigma_{n}^{2}}{N}\text{I}+\frac{\beta^{2}}{4N}({\text{H}}_{N}-{\bar{\text{H}}}_{N})\text{F}{\text{F}}^{T}({\text{H}}_{N}-{\bar{\text{H}}}_{N}), \end{array}\}$$

and **I** is the identity matrix. Then, the signal to interference plus noise ratio (SINR) for data stream *k* using codeword *i* is denoted as *γ*_*ki*_, which can be represented for the dispersive channel as

2.14 $$\gamma_{ki}=\frac{\text{signal}}{\text{IChI}+\text{MAI}+\text{additive noise}+\text{clipping noise}},$$

where

2.15 $$\begin{array}{rl} & \text{signal}=r^{2}\beta^{2}h_{k}^{2}[0]P_{\text{max}}^{2},\\ & \text{additive noise}=\sigma_{\text{n}}^{2}{\text{w}}_{i}^{T}{\text{w}}_{i},\\ & \text{clipping noise}=\sigma_{\text{clip}}^{2}{\text{w}}_{i}^{T}{\text{w}}_{i}\\ \;\text{and}\; & \text{IChI+MAI}=r^{2}\beta^{2}{\text{w}}_{i}{\text{A}}_{k}\Sigma_{d}{\text{A}}_{k}^{T}{\text{w}}_{i}^{T}-2r\beta {\text{e}}_{k}{\text{A}}_{k}{\text{w}}_{i}^{T}+\frac{2M_{k}-1}{6M_{k}-6}, \end{array}\}$$

where **Σ**_*d*_ = *E*{**dd**^*T*^}, **e**_*k*_ = *E*{*d*_*k*_**d**^*T*^}, and ${\text{A}}_{k}=({\text{H}}_{N}-{\bar{\text{H}}}_{N})\sum_{\ell }h_{k}[\ell ]{\text{I}}^{\left(\ell \right)}{\left({\text{H}}_{N}-{\bar{\text{H}}}_{N}\right)}^{T}$. Therefore, the BER expression for data stream *k* using the *i*th codeword can be approximated as

2.16 $$\text{BER}(\gamma_{ki},M_{k})\approx \frac{M_{k}-1}{2M_{k}\log_{2}M_{k}}\cdot \text{erfc}\left(\sqrt{\frac{3\gamma_{ki}}{\sqrt{2}(M_{k}^{2}-1)}}\right).$$

### (d) DC-reduced Hadamard-coded modulation

For indoor VLC systems, transmitting a signal with a high DC value can provide a higher illumination, which is not a waste of power. However, lowering the DC value reduces the magnitude of the signal, which prevents the signal from experiencing peak radiation power clipping. In [23], a modulation scheme that reduces the DC value, called DC-reduced HCM (DCR-HCM), is introduced. The average optical power is reduced by sending (**x** − min **x**) instead of **x**. Therefore, the DC level varies over symbols. At the receiver, the DC value removed before transmission does not affect the demodulation of the data.

By reducing the DC value, the maximum number of levels in the DCR-HCM transmitted signal is much lower than that for regular HCM, which simplifies the hardware structure and reduces the effects of the nonlinearity of the LEDs and their drivers on the transmitted signals. The maximum numbers of levels in HCM and DCR-HCM are denoted as *μ*_HCM_ and *μ*_DCR_, respectively, which can be calculated as

2.17 $$\begin{array}{rl} & \mu_{\text{HCM}}=N(M-1)+1\\ \;\text{and}\; & \mu_{\text{DCR}}=\frac{NM}{4}. \end{array}\}$$

**Figure 4.**
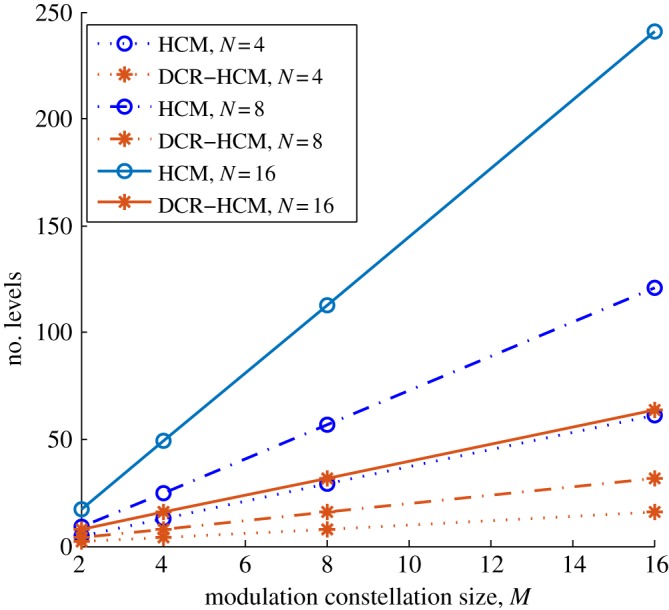
Numbers of levels for HCM and DCR-HCM signals. (Online version in colour.)

Figure 4 shows the maximum number of levels in the transmitted signal using HCM and DCR-HCM, calculated using (2.17). They both increase as the modulation constellation size increases, as expected. The number of levels in DCR-HCM is about 70% less than using HCM when a Hadamard code with the same order is used. For example, the number of levels in HCM using *N* = 4 is similar to that of DCR-HCM using *N* = 16 over different modulation constellation sizes, which implies that they can use hardware with similar complexity. However, because of the larger *N*, 16-DCR-HCM can support 12 more data streams than 4-HCM when using this same hardware structure.

## 3. Hadamard-coded modulation MAC layer design

HCM provides an attractive modulation scheme for multiuser indoor VLC systems, especially for IoT networks. In this section, the proposed Hadamard codeword assignment scheme and the effects of using multiple lamps to transmit the modulated signal are discussed.

### (a) Single lamp scenario

In this section, we consider a simple case where one lamp serves multiple users, and explore the use of HCM as a multiple access scheme.

#### (i) Codeword assignment

We assume that the users could have bandwidths that are limited to different values, and, therefore, an algorithm that appropriately assigns codewords to users is required. The Hadamard codewords with low-bandwidth requirements should be attributed to users that have low sampling abilities, to avoid IChI caused by the bandlimiting filters. When assigning high bandwidth codewords to low-bandwidth users, severe IChI could be introduced.

One codeword is assigned per user if either the throughput requirements are relatively low or the number of users is the same as (or greater than) the number of codewords. Let *a*_*ki*_ ∈ {0, 1} be an indicator function describing the status of codeword *i*: if *a*_*ki*_ = 1, codeword *i* is assigned to user *k*, and otherwise it is not. **A** is the *K* × (*N* − 1) matrix formed by elements *a*_*ki*_. In this section, codewords are optimally assigned based on their bandwidth and the SINR of each user, given in (2.14). To optimize the codeword assignment fairly, we maximize the minimum data rate over all the users, which can be described as

3.1 $$\begin{array}{rl} & \max_{\text{A},R_{s},M_{k}}\min_{k}\;\sum_{i}a_{ki}R_{s}\log_{2}M_{k},\\ \;\text{such that}\; & \sum_{k=1}^{K}a_{ki}\leq 1,\;\forall i\;\sum_{i=1}^{N-1}a_{ki}=1,\;\forall k\;\text{BER}(\gamma_{ki},M_{k})\leq {\text{BER}}_{\text{max}},\;\forall k, \end{array}\}$$

which is an integer optimization process since each codeword can only be assigned to one user. To guarantee that all the codewords are assigned no more than once, we must impose optimization constraints. For each user, the modulation constellation size can be changed to improve the throughput. The largest modulation constellation size that guarantees an acceptable BER, denoted BER_max_, based on the SINR at the receiver can be used, as shown in (3.1) as the last constraint. Integer optimization is time-consuming and inefficient; a heuristic searching scheme, such as a genetic algorithm, is often used to find the optimized solution, the process of which is standard and can be found in [24]. The steps used to formulate the genetic algorithm for this problem are given in the appendix.

When the number of users, *K*, is less than *N* − 1, more than one codeword can be assigned to one user for a higher transmission throughput. To optimize the throughput fairly, the users that have poor channel conditions can be assigned more codes for a better performance. This optimization process can be modelled as (3.1) but without the constraint that $\sum_{i=1}^{N-1}a_{ki}=1$. To guarantee that all the codewords are assigned once, we still impose the optimization constraint, $\sum_{k=1}^{K}a_{ki}\leq 1$, shown in (3.1). A genetic algorithm can again be used to find a near-optimal solution.

#### (ii) Numerical results

**Figure 5.**
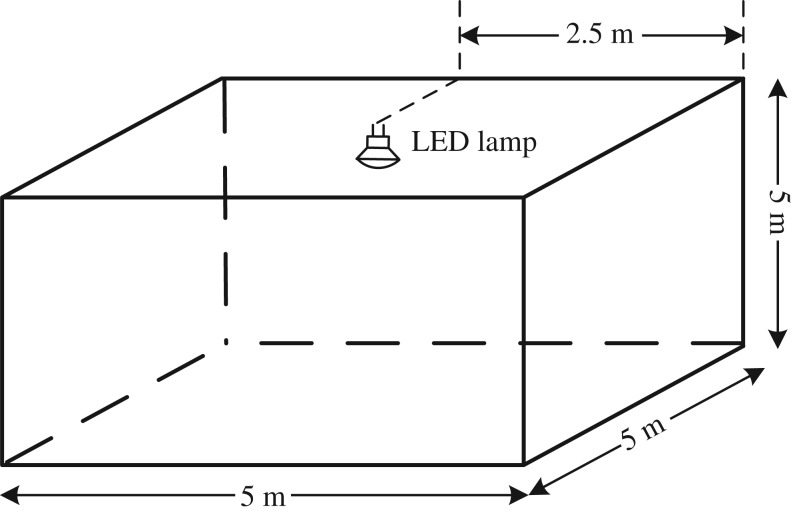
One lamp indoor environment used to generate the numerical results in this section.

In this section, we provide results for the case where one LED lamp transmits the signals intended for all users, the geometry of which is shown in figure 5. Unless otherwise noted, the parameters used to obtain the numerical results are shown in table 2, which is typical for indoor spaces and often used as a benchmark [12]. The test environment is a 5 m × 5 m × 3 m empty room. For the single lamp case, different users are assumed to have different bandwidths, as denoted in the table. The plots in this section are the results of a 50-trial Monte Carlo simulation assuming the users are randomly chosen and uniformly distributed in the room. All results shown in this paper are obtained by using the analytical expressions presented above. Considering the users’ different bandwidths, a bit loading algorithm is applied to maximize the data rate; the largest modulation constellation size is selected for each codeword based in the codeword’s SINR and *M*-PAM’s minimum SINR requirements.

**Table 2.** Parameters used for numerical results.

peak optical power limit, *P*_max_	5 W
size of Hadamard matrix, *N*	4, 8, 16, 32
noise spectral density, *N*_0_	1.5 × 10^−12^ mW Hz^−1^
acceptable BER, BER_max_	10^−4^
bandwidth for users 1, 3, 5, 7, 16, 18, 20, and 22	160 MHz
bandwidth for users 9, 11, 13, 15, 24, 26, 28, and 30	160 MHz
bandwidth for users 8, 10, 12, 14, 23, 25, 27 and 29	80 MHz
bandwidth for users 4, 6, 19, and 21	40 MHz
bandwidth for user 2, 17	20 MHz

With the help of forward error control (FEC) coding, an uncoded BER of 10^−4^ is usually needed, as listed in table 2. Relaxing this constraint does not affect the optimization process; a more relaxed BER constraint allows transmitting a higher data rate since a larger modulation constellation size and a faster symbol rate can be used. However, FEC codes that provide an acceptable transmission quality with a more relaxed BER constraint are more complex and not currently suitable for simple IoT devices. All of our results use BER_max_ = 10^−4^.

**Figure 6.**
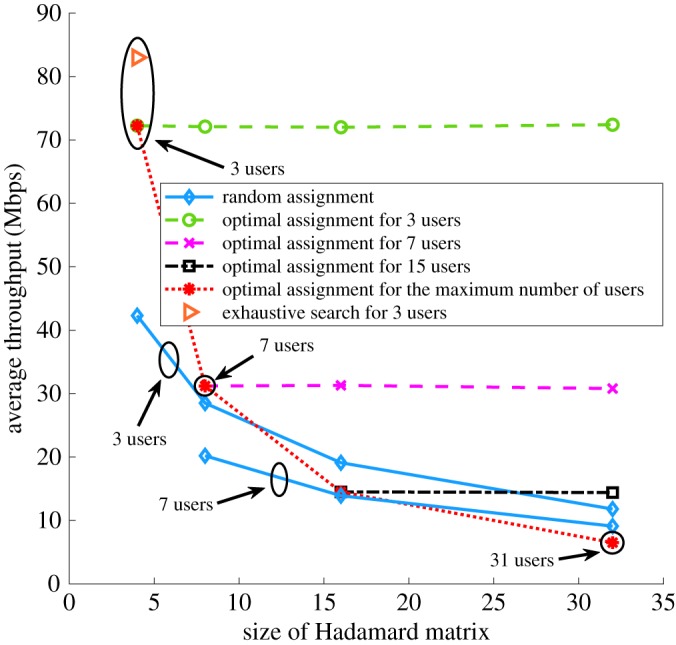
Average per-user data rate comparison of randomly and optimally choosing the codewords assigned to users. (Online version in colour.)

Figure 6 shows the average data rate over the users when the codewords are optimally assigned compared to when they are randomly assigned to the users, assuming exactly one codeword is assigned per user. As the size of the Hadamard matrix increases, more users can be supported. When randomly assigning codewords to users, a larger Hadamard matrix is more likely to assign a high bandwidth codeword to a user with low bandwidth, which introduces severe IChI. Therefore, as shown in figure 6, for a random codeword assignment the average data rate decreases for the same number of users as the size of the Hadamard matrix increases. When optimally assigning the codewords, increasing the size of the Hadamard matrix does not affect the average data rate since the same codewords are assigned to the users. Comparing random and optimal codeword assignments, the average data rate of the optimal codeword assignment is approximate twice that of randomly choosing the codewords when three and seven users are supported.

In this paper, the genetic algorithm used to obtain the simulation results provides a trade-off between optimality and feasibility in solving the optimization problem. In figure 6, we compare the performance using the genetic algorithm and an exhaustive search that guarantees the globally optimal solution for the simplest case of three users and an *N* = 4 Hadamard matrix. The average throughput using an exhaustive search is about 16% higher than using the genetic algorithm. Although an exhaustive search finds a better solution, it is impractical for a real-time implementation involving a large number of users due to the unacceptable running time.

The throughput can be enhanced by assigning multiple codewords to users. Table 3 shows the average throughput achieved when assigning multiple codewords to each user as a function of the number of users in the room. Based on the channel conditions of the users, a different number of codewords can be optimally assigned to each user. Compared with using a single codeword per user, assigning multiple codewords can improve the throughput by about 10% when the number of users is much smaller than the size of the Hadamard code. As the number of users increases, a smaller number of available codewords can be assigned to users; in this case, the throughput achievable when assigning multiple codewords per user is similar to assigning a single codeword per user. The reason the improvement is not greater is that the system continues to operate under a fixed maximum power constraint, so that increasing the number of codewords used diminishes the signal power available for other codewords.

**Table 3.** Average throughput using single versus multiple codewords per user for *N* = 16.

throughput (Mbps)	3 users	4 users	5 users	6 users	7 users
single codeword per user	72.1	53.4	44.6	35.6	31.3
multiple codewords per user	78.5	59.3	47.3	37.9	32.4

### (b) Effect of multiple lamps

In a typical indoor VLC system, there are several LED lamps working as light sources and transmitters; thus, the effects of using multiple lamps need to be considered. In this section, we assume that the NLOS portion of the channel impulse response is negligible. We focus on the overlap of the LOS components of the signals originating at the various lamps.

#### (i) Performance analysis

**Figure 7.**
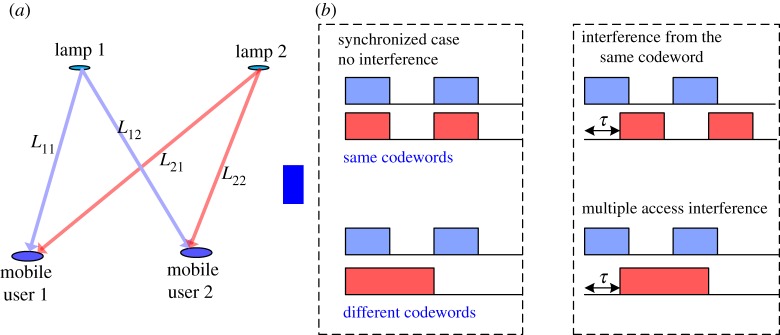
(*a*) Illustration of a two-lamp scenario, (*b*) illustration of multiple access interference and inter-chip interference. (Online version in colour.)

We assume that each user can be served by *Q* lamps that send the same data synchronously and that each user is synchronized to the lamp closest to it. The signals emitted from the lamps are received and combined at the receiver. A two-user two-lamp example is shown in figure 7*a*. The propagation distance between user *k* and lamp *q* is denoted as *L*_*qk*_. Because the propagation distances to the various lamps for a particular user are different, the propagation delay is different, resulting in MAI and IChI; the codewords are no longer orthogonal at the receiver due to the propagation delay. MAI and IChI are caused by the cross-correlation due to the signal arrival time differences. This phenomenon is illustrated in figure 7*b*. After HCM demodulation and MMSE filtering, the estimate of the data for user *k* can be represented as

3.2 $${\hat{d}}_{k}=r\beta h_{ik}g_{kk}(0)\text{d}{\text{w}}_{k}^{T}+r\beta \text{d}\sum_{q\neq i}^{Q}h_{qk}\text{G}\left(\tau_{qk}\right){\text{w}}_{k}^{T}+\tilde{\text{n}}{\text{w}}_{k}^{T}+{\tilde{\text{n}}}_{\text{clip}}{\text{w}}_{k}^{T},$$

where *h*_*ik*_ represents the channel gain from lamp *i* to user *k*. **G**(*τ*_*qk*_) is the cross-correlation matrix as a function of the signal arrival time difference, *τ*_*qk*_, which is represented as

3.3 $$\text{G}(\tau_{qk})=\left(\begin{array}{cccc} 0 & g_{12}(\tau_{qk}) & \cdots & g_{1N}(\tau_{qk})\\ g_{21}(\tau_{qk}) & 0 & \cdots & g_{2N}(\tau_{qk})\\ \vdots & \vdots & \ddots & \vdots \\ g_{N1}(\tau_{qk}) & g_{N2}(\tau_{qk}) & \cdots & 0 \end{array}\right).$$

The elements of the cross-correlation matrix are calculated as

3.4 $$g_{ij}(\tau_{qk})=\frac{T_{s}}{T_{c}}\sum_{n=0}^{\left(N-1)(T_{c}/T_{s}\right)}c_{i}(nT_{s})c_{j}(nT_{s}+\tau_{qk}),$$

where *T*_*s*_ represents the sampling period at the receiver. *T*_*c*_ is the chip period of the codeword with the highest bandwidth, which is 1/(*NR*_0_), and *R*_0_ is the transmitted symbol rate.

The propagation delay from LED *q* to user *k*, *τ*_*qk*_, affects the MMSE filter at the receiver. If user *k* is synchronized to *i*th LED, then $\tau_{qk}=(L_{qk}-L_{ik})/C$, where C is the speed of light. The MMSE filter for user *k* becomes

3.5 $${\text{w}}_{k}=r\beta \left({\text{e}}_{k}\left(\sum_{q\neq i}^{Q}h_{qk}\text{G}(\tau_{qk})\right)-h_{ik}{\text{e}}_{k}\right){\left(\text{Z}+\sigma_{n}^{2}\text{I}\right)}^{-1},$$

where **Z** is defined as

3.6 $$\text{Z}=r^{2}\beta^{2}\left(h_{ik}^{2}\Sigma_{d}+h_{ik}\left(\sum_{q\neq i}^{Q}h_{qk}\text{G}(\tau_{qk})\right)\Sigma_{d}+\left(\sum_{q\neq i}^{Q}h_{qk}{\text{G}}^{T}(\tau_{qk})\right)\Sigma_{d}\left(\sum_{q\neq i}^{Q}h_{qk}\text{G}(\tau_{qk})\right)\right).$$

Therefore, the peak SINR for user *k* synchronized to the *i*th lamp can be approximated as

3.7 $$\gamma_{ik}=\frac{\text{signal}}{\text{MAI}+\text{IChI}+\text{additive noise}+\text{clipping noise}},$$

where

3.8 $$\begin{array}{r} \begin{array}{rl} \text{signal} & =r^{2}\beta^{2}h_{ik}^{2}P_{\text{max}}^{2},\\ \text{additive noise} & =\sigma_{\text{n}}^{2}{\text{w}}_{k}^{T}{\text{w}}_{k},\\ \text{clipping noise} & =\sigma_{\text{clip}}^{2}{\text{w}}_{k}^{T}{\text{w}}_{k},\\ \text{MAI}+\text{IChI} & =\frac{2M_{k}-1}{6M_{k}-6}-2r\beta h_{ik}{\text{w}}_{k}{\text{e}}_{k}-2r\beta {\text{w}}_{k}\left(\sum_{q\neq i}^{Q}h_{qk}{\text{G}}^{T}(\tau_{qk})\right){\text{e}}_{k}^{T}+r^{2}\beta^{2}h_{ik}^{2}{\text{w}}_{k}\Sigma_{d}{\text{w}}_{k}^{T}\\ & \;+2r^{2}\beta^{2}h_{ik}{\text{w}}_{k}\left(\sum_{q\neq i}^{Q}h_{qk}{\text{G}}^{T}(\tau_{qk})\right)\Sigma_{d}{\text{w}}_{k}^{T}\\ & \;+r^{2}\beta^{2}{\text{w}}_{k}\left(\sum_{q\neq i}^{Q}h_{qk}{\text{G}}^{T}(\tau_{qk})\right)\Sigma_{d}\left(\sum_{q\neq i}^{Q}h_{qk}\text{G}(\tau_{qk})\right){\text{w}}_{k}. \end{array} \end{array}$$

#### (ii) Numerical results

**Figure 8.**
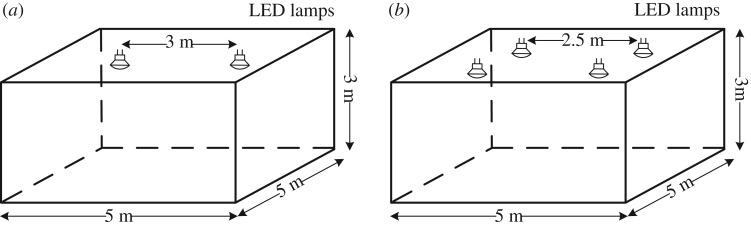
Indoor environments used to generate the numerical results in this section. (*a*) Two-lamp scenario, (*b*) four-lamp scenario.

The impact on system performance of multiple lamps transmitting user signals is considered in this section. The users’ bandwidths are now assumed to be equal and large enough for the chip rate in order to highlight the arrival time difference between received lamp signals as the primary degradation to the system performance. For our simulation results, we test two scenarios, based on two-lamps and four-lamps, the room geometries for which are illustrated in figure 8*a*,*b*, respectively.

**Figure 9.**
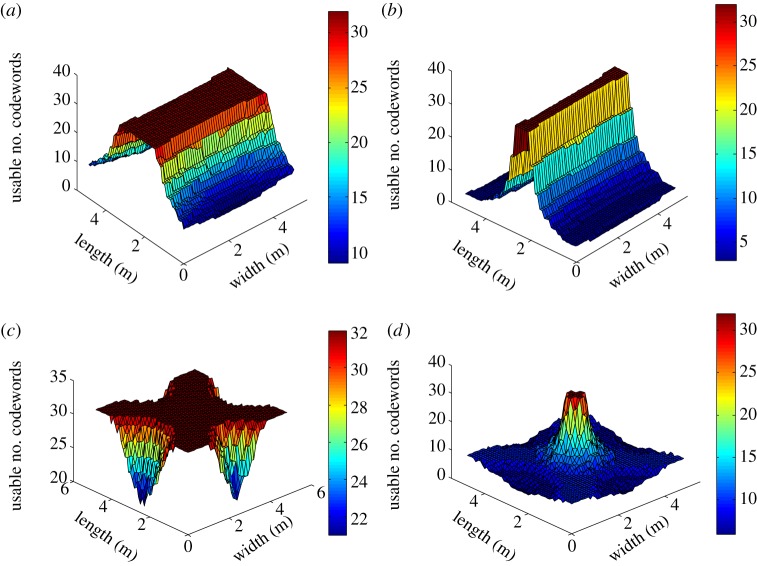
Number of usable codewords in an indoor space for *N* = 32, and *T*_*c*_ = 16.6 ns. (*a*) Two-lamp scenario using 2-PAM, (*b*) two-lamp scenario using 4-PAM, (*c*) four-lamp scenario using 2-PAM, (*d*) four-lamp scenario using 4-PAM. (Online version in colour.)

The users located at the various locations in the room have different time of arrival differences between the signals sent by the multiple lamps. Thus, the MAI and IChI vary significantly over the indoor space. Since each codeword has a specific tolerance to MAI and IChI, some of the codewords cannot provide an acceptable SINR for the modulation constellation used. Figure 9 shows the spatial distribution of the numbers of codewords that can be used for *N* = 32. In figures 9*a*,*c*, when 2-PAM is used, most of the indoor area can potentially use all 31 codewords. When 4-PAM is applied, however, only areas in the room with a relatively short time of arrival difference can use all 31 codewords, as shown in figure 9*b*,d. The edges and corner areas of the room have larger time of arrival differences than the centre of the room; only about five codewords can be supported there and still achieve an acceptable BER for 4-PAM.

**Figure 10.**
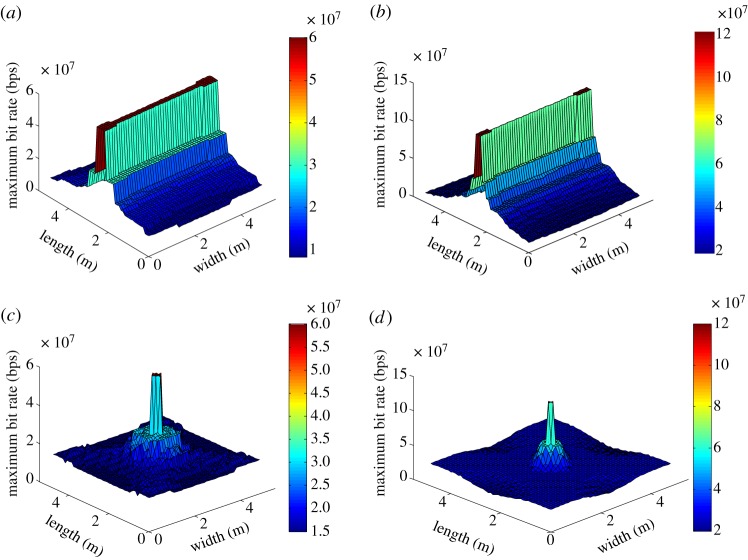
Spatial distribution of the maximum throughput in the room with a fixed symbol rate: (*a*) *Q* = 2 and *T*_*s*_ = 1 Msps, (*b*) *Q* = 2 and *T*_*s*_ = 2 Msps, (*c*) *Q* = 4 and *T*_*s*_ = 1 Msps (*d*) *Q* = 4 and *T*_*s*_ = 2 Msps. (Online version in colour.)

The distribution of the maximum total throughput in the indoor space using the two-lamp and four-lamp scenarios is illustrated in figure 10. For this result, the modulation constellation size is optimally chosen at each specific user location. The throughput is calculated as the usable number of codewords times the data rate per codeword. Comparing figure 10*a*,*b* that use different symbol rates in the two-lamp scenario, we observe that a lower symbol rate results in a more uniform throughput distribution due to the lower MAI and IChI. The behaviour of the four-lamp scenario, the results of which are shown in figure 10*c*,*d*, is similar to the two-lamp case.

## 4. Conclusion

In this paper, we propose an indoor multiuser VLC system design based on HCM. A fast Walsh–Hadamard transform is used to efficiently modulate the intended data for multiple users on orthogonal Hadamard codewords. To reduce the nonlinear distortion caused by the peak transmitted power constraint of the LEDs, a DC-reduced HCM can be used. Compared with the standard HCM, DCR-HCM has a lower signal magnitude and a smaller number of signal magnitude levels, which can simplify the hardware structure. The codewords in the Hadamard code family require different bandwidths. For practical applications, such as IoT, users often operate with different maximum bandwidths due to their restricted sampling rates. This paper proposes to optimally assign the codewords to minimize the ISI caused by the bandlimited users’ devices. Optimally assigning codewords to users can more than double the throughput. When assigning multiple codewords to one user, the throughput can be further enhanced by about 10% compared to using only one codeword per user, for the parameters given. Considering the multi-lamp scenario, multiple access interference and inter-chip interference for each codeword are different. Based on the indoor environment and users’ locations, different numbers of codewords can be acceptably used. From the simulation results, portions of the indoor space with relatively small differences in the time of arrival of the received signals can use a larger number of codewords and result in a higher throughput.

## Data accessibility

This article has no additional data.

## Appendix

This appendix lists the steps needed to implement the genetic algorithm to find the optimized codeword assignment; more detail can be found in [24]. The pseudo-code is shown in algorithm 1. When the users’ location and bandwidth are given, a near-optimal codeword assignment to maximize the throughput can thus be found.
